# LINC00908 negatively regulates microRNA-483-5p to increase TSPYL5 expression and inhibit the development of prostate cancer

**DOI:** 10.1186/s12935-019-1073-x

**Published:** 2020-01-09

**Authors:** Li Fan, Hai Li, Yun Zhang

**Affiliations:** Department of Urology, China-Japan Union Hospital of Jilin University, No. 126, Xiantai Street, Changchun, 130033 Jilin People’s Republic of China

**Keywords:** Prostate cancer, LINC00908, microRNA-483-5p, TSPYL5, Proliferation, Migration, Invasion, Apoptosis

## Abstract

**Background:**

Accumulating evidence has associated aberrant long non-coding RNAs (lncRNAs) with various human cancers. This study aimed to explore the role of LINC00908 in prostate cancer (PCa) and its possible underlying mechanisms.

**Methods:**

Microarray data associated with PCa were obtained from the Gene Expression Omnibus (GEO) to screen the differentially expressed genes or lncRNAs. Then, the expression of LINC00908 in PCa tissues and cell lines was detected by reverse transcription-quantitative polymerase chain reaction (RT-qPCR). The localization of LINC00908 in PCa cells was examined by fluorescence in situ hybridization (FISH). The relationship among LINC00908, microRNA (miR)-483-5p, and TSPYL5 was detected by bioinformatics analysis, dual-luciferase reporter assay, RNA pull-down, RNA binding protein immunoprecipitation (RIP), and FISH assays. Cell biological behaviors were assessed after the expression of LINC00908, miR-483-5p, and TSPYL5 was altered in PCa cells. Lastly, tumor growth in nude mice was evaluated.

**Results:**

Poorly expressed LINC00908 was witnessed in PCa tissues and cells. LINC00908 competitively bound to miR-483-5p to up-regulate the TSPYL5 expression. Overexpression of LINC00908 resulted in reduced PCa cell proliferation, migration and invasion, and promoted apoptosis. Additionally, the suppression on PCa cell proliferation, migration and invasion was induced by up-regulation of TSPYL5 or inhibition of miR-483-5p. In addition, in vivo experiments showed that overexpression of LINC00908 inhibited tumor growth of PCa.

**Conclusion:**

Overall, LINC00908 could competitively bind to miR-483-5p to increase the expression of TSPYL5, thereby inhibiting the progression of PCa. Therefore, LINC00908 may serve as a novel target for the treatment of PCa.

## Background

Prostate cancer (PCa) is one of the leading causes of cancer-related deaths in men across the globe [1]. It is well-known that the action of dihydrotestosterone can affect the progression of PCa via androgen receptors [2]. In recent years, the world has witnessed significant improvements in the prevention and treatment approaches of PCa such as androgen deprivation therapy, which is still the mainstay therapy for PCa [3]. Interestingly, gene therapy has emerged as an appealing therapeutic method for prostate diseases, but remains to be underdeveloped [4], therefore more in-depth investigation is needed to reveal the underlying cancer mechanisms. Fortunately, long non-coding RNAs (lncRNAs) have been shown to play important roles in the initiation and tumorigenesis of PCa [5, 6], and could be crucial to identify and provide novel targets to protect against the progression of PCa.

LncRNAs are a family of RNAs involved in various biological processes during every stage of life and usually regarded as potential cancer targets [7]. For example, lncRNA insulin growth factor 2 antisense (IGF2AS) acts as a tumor suppressor in PCa and its overexpression results in inhibition of cancer cell proliferation and progression [8]. A recent study showed that lncRNA human chorionic gonadotropin 11 (HCG11) was poorly expressed in PCa and related to the cancer pathologic features [9]. Additionally, the overexpression of lncRNA growth arrest-specific transcript 5 (GAS5) has been previously demonstrated to suppress cell migration and invasion in PCa [10]. Based on the web-available microarrays prior to our study, LINC00908 has been revealed to be significantly down-regulated in PCa. Moreover, it has also been demonstrated that lncRNAs may possess the ability to antagonize the post-transcriptional regulation of microRNAs (miRNAs or miRs) in the expression of genes, hence playing a vital role in human disease development [11]. MiRNAs are a class of small ncRNAs with 18–24 nucleotides in length, which are involved in the regulation of cellular processes [12]. Additionally, miRNAs have been demonstrated to potentially target a variety of molecules and to refine the output of proteins [13]. It has been demonstrated that miRNAs play important roles in multiple cancers. For instance, overexpression of miR-15b-5p has been previously documented to accelerate the progression of PCa [14]. Interestingly, another study also demonstrated that downregulation of miR-483-5p causes suppression of PCa cell growth and invasion [15]. Testis-specific protein, Y-encoded-like 5 (TSPYL5) was identified as a target of miR-483-5p by the biological prediction RNA22 website (https://cm.jefferson.edu/rna22/). TSPYL5 has been assumed to exert a tumor-suppressive function, whose hypermethylation is frequently linked with various types of human disease and cancers [16]. Moreover, diminished expression of TSPYL5 protein in advanced stages of PCa have also been regarded as a putative biomarker of PCa development [17]. Thus, the current study aims to explore the underlying molecular mechanisms of the interplay between LINC00908, TSPYL5, and miR-483-5p in the development of PCa in a bid to discover a promising competitive new target for PCa treatment.

## Materials and methods

### Ethics statement

The current study was approved by the Ethics Committee of China-Japan Union Hospital of Jilin University and all experiment protocols abided by the Declaration of Helsinki. Signed informed consents were obtained from all participants prior to the study. All animal experiments were approved by the Animal Ethics Committee of China-Japan Union Hospital of Jilin University, and all measures were undertaken to minimize the usage of animals as well as their suffering.

### Microarray-based analysis

PCa expression profiles were retrieved from the Gene Expression Omnibus (GEO) database (https://www.ncbi.nlm.nih.gov/geo/). Limma package of the R language was applied for differential gene expression analysis and the *p* value was corrected using the false discovery rate (FDR) method. The threshold for screening differentially expressed genes in PCa was set as |log fold change (FC)| > 1, *p*-value < 0.05, and the box plot was plotted according to the obtained values.

### Study subjects

A total of 55 patients with PCa were selected from the China-Japan Union Hospital of Jilin University between April 2014 and May 2015. There were 27 patients aged < 60 years and 28 aged ≥ 60 years; 21 with a tumor size < 3 cm and 34 with tumor size ≥ 3 cm. The paracancerous tissues (3 cm away from the PCa tissues) were collected and regarded as the controls. The included cases were categorized according to tumor-nodes-metastasis (TNM) classification criterion, with 14 cases in stage I, 10 cases in stage II and 31 cases in stage III. None of the included patients underwent chemotherapy or radiotherapy prior to the surgery.

### Cell culture

Human PCa cell lines VCaP, LNCaP, DU-145, PC-3, and human prostate epithelial cell line RWPE-1 were purchased from the Cell Bank of American Type Culture Collection (ATCC; Manassas, VA, USA). Subsequently, reverse transcription quantitative polymerase chain reaction (RT-qPCR) was performed to detect the expression of LINC00908 in the aforementioned cell lines. The cell line exhibiting the lowest LINC00908 expression was selected for subsequent experimentation. Cells were then cultured in Roswell Park Memorial Institute (RPMI) 1640 medium containing 10% serum at 37 °C with 5% CO_2_. The medium was renewed every 2–3 days depending on cell growth. When reaching approximately 80–90% confluence, the cells were subcultured accordingly.

### Cell treatment

According to the known sequences of LINC00908, miR-483-5p and TSPYL5 from National Center for Biotechnology Information (NCBI), Shanghai Sangon Biotechnology Co., Ltd. (Shanghai, China) was commissioned to construct overexpression (oe)-LINC00908 and oe-TSPYL5 vectors or synthesize the sequences of small interfering RNA (si)-TSPYL5, miR-483-5p mimic and miR-483-5p inhibitor. Human PCa cell line LNCaP displaying the lowest LINC00908 expression was selected for subsequent experiments. LNCaP cells at passage 3 were trypsinized, seeded into 24-well plates and allowed to grow into a monolayer, after which the culture medium was removed. Next, the cells were transfected with oe-negative control (NC) (transfection with pcDNA3 empty vectors), oe-LINC00908 (transfection with pcDNA3 plasmids of overexpressed LINC00908), inhibitor NC (transfection with inhibitor NC sequences, serving as the control of miR-483-5p inhibitor), miR-483-5p inhibitor (transfection with miR-483-5p inhibitor sequences), oe-TSPYL5 (transfection with pcDNA3 plasmids of overexpressed TSPYL5), oe-LINC00908 + miR-483-5p mimic (co-transfection with pcDNA3 plasmids of overexpressed LINC00908 and miR-483-5p mimic sequences), and miR-483-5p inhibitor + si-TSPYL5 (co-transfection with miR-483-5p inhibitor and TSPYL5 silencing sequences).

The PCa cells were plated into 6-well plates 24 h prior to transfection. When cell density reached 70–80%, cell transfection was performed in accordance with the manufacturer’s instructions of the Lipofectamine 2000 reagent (11668-019, Invitrogen Inc., Carlsbad, CA, USA). The transfected cells were subsequently cultured in a 5% CO_2_ incubator at 37 °C for 6–8 h. With the replacement of complete culture medium, the culture was conducted for another 24–48 h.

### Fluorescence in situ hybridization (FISH)

The subcellular localization of LINC00908 in PCa cells was predicted using a biological prediction lncATLAS website (http://lncatlas.crg.eu/) and identified with FISH assay according to the manufacturer’s instructions of Ribo™ lncRNA FISH Probe Mix (Red) (RiboBio Co., Ltd., Guangzhou, Guangdong, China). Briefly, coverslips were placed on 24-well plates, with the cells inoculated at a density of 6 × 10^4^ cells/well. The coverslips were fixed with 1 mL 4% paraformaldehyde at room temperature after phosphate-buffered saline (PBS) rinsing. The cells were subsequently treated with 2 μg/mL protease K (Beijing Solabio Life Sciences Co., Ltd., Beijing, China), glycine and acetylation reagent, and then incubated with 250 μL prehybridization solution at 42 °C for 1 h. Next, 250 μL hybridization solution containing 300 ng/mL probe was added following removal of the prehybridization solution and then incubated at 42 °C overnight. The following day, the cells were washed three times with PBS-Tween-20 (PBST), followed by the addition of 4′,6-diamidino-2-phenylindole (DAPI) staining solution diluted by PBST at a ratio of 1:800 to the 24-well plate for nucleus staining for 5 min. After three PBST (3 min each time), the coverslips were sealed with an anti-fluorescence quencher. Finally, 5 different fields were randomly selected, observed and photographed under a fluorescence microscope (Olympus, Tokyo, Japan).

### RT-qPCR

Total RNA was extracted from the tissues according to the manufacturer’s instructions of Trizol method, while total RNA in transfected cells from each group was extracted using a miRNeasy Mini Kit I (217004, QIAGEN, Hilden, Germany). The primers of LINC00908, miR-483-5p, and TSPYL5 were designed and synthesized by Takara Holdings Inc. (Kyoto, Japan) (Table 1). The extracted RNA was reverse transcribed into complementary DNA (cDNA) using a PrimeScript RT kit (RR036A, Takara Holdings Inc., Kyoto, Japan). A fluorescent quantitative PCR operation was carried out in accordance with the manufacturer’s instruction of the SYBR^®^ Premix ExTaq™ II Kit (RR820A, Takara Holdings Inc., Kyoto, Japan). RT-qPCR was then performed on an ABI PRISM^®^ 7300 system (Prism^®^ 7300, Shanghai Kunke Instrument Equipment Co., Ltd., Shanghai, China). U6 was regarded as the internal reference for miR-483-5p, and β-actin was regarded as the internal reference for LINC00908 and TSPYL5. The relative expression of each gene was calculated using the 2^−ΔΔCt^ method.

**Table 1:** Primer sequences for RT-qPCR

Gene	Sequences (5′-3′)
LINC00908	F: CTATCCACGGACGCCTTCTC
	R: CTTGGTGTGTCCTCCCTTCC
miR-483-5p	F: AAGACGGGAGGAAAGAAGGGAG
	R: GTGCAGGGTCCGAGGTATTC
TSPYL5	F: CGTGGGAAGGCCGAAAAATG
	R: TCCCACCAGCTATGACCTGA
β-actin	F: CGGGATCCATGGATGATGATATCGCCGCGC
	R: CGGAATTCCTAGAAGCATTTGCGGTGGACG
U6	F: CGCTTCGGCAGCACATATACTA
	R: CGCTTCACGAATTTGCGTGTCA

*F* forward, *R* reverse

### Western blot analysis

Total protein was extracted from the cells using a radioimmunoprecipitation assay (RIPA) lysis buffer (R0010, Beijing Solarbio Science & Technology Co., Ltd., Beijing, China) containing phenylmethylsulfonyl fluoride. The cells were then incubated on ice for 30 min and centrifuged at 1200×*g* at 4 °C for 10 min, followed by the collection of supernatant containing protein for subsequent protein quantitation. An amount of 50 μg protein was dissolved in 2 × sodium dodecyl sulfate (SDS) loading buffer and boiled for 5 min at 100 °C. After that, the protein was transferred onto a polyvinylidene fluoride membrane after protein separation was performed with SDS-polyacrylamide gel electrophoresis (PAGE). The membrane was then blocked using 5% skim milk powder for 1 h at room temperature, followed by PBS rinsing for 2 min and overnight incubation at 4 °C with the primary antibodies: rabbit monoclonal antibodies to TSPYL5 (dilution ratio of 1:1000, ab203657) and matrix metalloproteinase (MMP)-2 (dilution ratio of 1:500, ab37150), as well as rabbit polyclonal antibodies to MMP-9 (dilution ratio of 1:1000, ab38898) and β-actin (dilution ratio of 1:1000, ab8227). All aforementioned antibodies were purchased from Abcam Inc. (Cambridge, MA, USA). After incubation, the samples were then washed three times with Tris-buffered saline-Tween (5 min/time) and further incubated with secondary antibody, horseradish peroxidase (HRP)-conjugated goat anti-mouse immunoglobulin G (IgG) (HA1003, Shanghai Yanhui Biotechnology Co., Ltd., Shanghai, China) for 1 h. Finally, the membrane was developed with enhanced chemiluminescence solution (808-25, Biomiga, San Diego, CA, USA) at room temperature for 1 min. The results were visualized with an exposure machine using the Wes automatic protein blot quantification analysis system. The relative protein expression was expressed as the ratio of gray value of the target protein band to that of β-actin protein band.

### Dual-luciferase reporter assay

Dual-luciferase reporter assay was applied to explore the binding sites between miR-483-5p and LINC00908, as well as to verify whether TSPYL5 was the direct target gene of miR-483-5p. PmirGLO Dual-Luciferase miRNA Target Expression Vector (Promega Corp., Madison, WI, USA) was utilized to construct the wild type-LINC00908 (Wt-LINC00908) and mutant-LINC00908 (Mut-LINC00908) vectors. The Wt-TSPYL5 and Mut-TSPYL5 vectors were constructed according to the sequence in which the 3′ untranslated region (UTR) of TSPYL5 mRNA binds to miR-483-5p. All plasmids were extracted in accordance with the manufacturer’s instructions of Omega plasmid miniprep kit (D1100-50T, Beijing Solabio Life Sciences Co., Ltd., Beijing, China). The cells were then seeded into a 6-well plate at a density of 2 × 10^5^ cells/well, and transfected in accordance with the aforementioned method after the cells adhered to the wall. The successfully transfected cells were collected after 48 h of culture. Luciferase activity was detected using a Dual-Luciferase Assay Kit (D0010, Beijing Solabio Life Sciences Co., Ltd., Beijing, China) on a Glomax 20/20 luminometer (E5311, Shaanxi Zhongmei Biotechnology Co., Ltd., Xi’an, Shaanxi, China).

### RNA pull-down assay

LNCaP cells were transfected with 50 nM biotin-labeled Wt-bio-miR-483-5p and Mut-bio-miR-483-5p for 48 h. The cells were then rinsed with PBS and incubated in specific lysate buffer (Ambion, Austin, TX, USA) for 10 min. Next, the lysates were incubated with M-280 streptavidin magnetic beads (S3762, Sigma-Aldrich Chemical Company, St Louis, MO, USA) pre-coated with RNase-free bovine serum albumin and yeast tRNA (TRNABAK-RO, Sigma-Aldrich Chemical Company, St Louis, MO, USA) at 4 °C for 3 h, rinsed twice with pre-chilled lysis buffer, thrice with low salt buffer, and once with high salt buffer. The bound RNA was purified with Trizol and the enrichment of LINC00908 in the obtained RNA was then detected using RT-qPCR.

### RNA binding protein immunoprecipitation (RIP)

The binding ability of LINC00908 to Argonaute 2 (AGO2) protein was detected using the RIP kits (Millipore, Bedford, MA, USA). Briefly, the cells were lysed using an equal volume of RIPA lysis buffer (P0013B, Beyotime Biotechnology Co., Shanghai, China) on an ice bath for 5 min and centrifuged at 4 °C at 14,000 r/min for 10 min, with the supernatant collected. A portion of the cell extract was used as an Input, while the other portion was incubated with antibodies for the co-precipitation experiment. In each co-precipitation reaction system, 50 μL of beads were resuspended in 100 μL of RIP wash buffer. Each group was added with 5 μg antibodies for binding purposes. The magnetic bead-antibody complex was then washed and resuspended in 900 μL RIP wash buffer, followed by overnight incubation with 100 μL cell extract at 4 °C. The samples were then placed on a magnetic pedestal to collect the magnetic bead-protein complexes. The samples and Inputs were then detached with proteinase K to extract RNA, which was used for RT-qPCR detection. The antibodies used for RIP were AGO2 (ab32381, dilution ratio of 1:50, Abcam Inc., Cambridge, MA, USA), while IgG (dilution ratio of 1:100, ab109489, Abcam Inc., Cambridge, MA, USA) was used as the NC.

### 5-ethynyl-2′deoxyuridine (EdU) staining

Each cell culture plate was added with EdU solution, incubated at room temperature for 2 h, and rinsed with PBS. Cells were then fixed with 40 g/L paraformaldehyde for 30 min, incubated with glycine solution for 5 min, and then rinsed with PBS containing 0.5% Triton X-100. Next, nuclear staining incubation was performed with the addition of Apollo^®^ staining solution under dark conditions at room temperature for 30 min, followed by two washes with formaldehyde and PBS respectively. Finally, Hoechst3334 staining solution was added to the cells at room temperature under dark conditions and the cells were further incubated for 30 min, followed by the observation of stained cells under a fluorescence microscope. Three fields of view were randomly selected and the EdU stained cells (proliferating cell) and Hoechst3334 stained cells (total cells) were subsequently counted using the following formula: the cell proliferation rate = the number of proliferating cells/total cells × 100%.

### Flow cytometry

After 48 h of transfection, the cells were detached with 0.25% trypsin, centrifuged at 1000 r/min at 4 °C for 5 min, and then centrifuged again at 1000 r/min for 5 min. After discarding the supernatant, the cells were fixed with pre-cooled 70% ethanol at 4 °C overnight and subsequently centrifuged at 1000 r/min for 5 min. Following incubation with 10 μL RNase at 37 °C for 5 min, the cells were then stained with 1% propidium iodide (PI; 40710ES03, Shanghai Qcbio Co., Ltd., Shanghai, China) in dark conditions for 30 min. The cell cycle distribution in connection with the detection of red fluorescence at an excitation wavelength of 488 nm was recorded using a flow cytometer (FACSCalibur, BD, FL, NJ, USA).

After 48-h transfection and detachment using ethylenediaminetetraacetic acid-free trypsin, the cells were centrifuged at 1000 r/min at 4 °C for 5 min. Subsequently, the cells were subjected to another centrifugation at 1000 r/min for 5 min. After discarding the supernatant, cell apoptosis was detected using an Annexin V-fluorescein isothiocyanate (FITC)/PI apoptosis detection kit (CA1020, Beijing Solarbio Life Sciences Co., Ltd., Beijing, China). The cells were washed with binding buffer and re-suspended in a mixture of Annexin-V-FITC and binding buffer at a ratio of 1:40, followed by the addition of a mixture of PI and binding buffer at a ratio of 1:40. Finally, cell apoptosis was detected using flow cytometry.

### Transwell assay

After the cells were transfected for 48 h, Matrigel (356234, BD Biosciences, San Jose, CA, USA) was dissolved overnight at 4 °C and diluted at a ratio of 1:10 with serum-free medium. Matrigel was then added to the apical chamber of Transwell chamber with 50 μL each well and placed in a culture incubator for 30 min. The cells were detached, rinsed thrice with serum-free medium, counted and dispersed into a cell suspension. Matrigel was washed once with serum-free medium, and the cell suspension was seeded into the apical chamber containing serum-free medium at a density of 1 × 10^5^ cells/mL, while medium containing 10% fetal bovine serum (FBS) was added to the basolateral chamber. The Transwell chamber was then placed in a culture plate and incubated at 37 °C for 24 h. After incubation, the Transwell chamber was washed twice with PBS (5 min each time) and fixed with 5% glutaraldehyde at 4 °C, stained with 0.1% crystal violet for 30 min, rinsed twice with PBS and observed under a microscope. The number of cells passing through Matrigel in each group was used as an indicator to evaluate its invasion ability.

### Scratch test

The cells at the logarithmic growth phase were seeded into 24-well plates at a density of 8 × 10^4^ cells/well, with three duplicates set for each group. Subsequently, a 10 μL sterilization tip was used for vertical scratching on the 24-well plate, and then the cells were rinsed 2–3 times with serum-free medium. The number of migrated cells on the scratched surface was counted under 5 randomly selected fields under a phase-contrast microscope.

### Tumor xenograft in nude mice

Thirty specific pathogen-free (SPF) nude mice were housed under constant temperature in sterile conditions. Stably transfected LNCaP cell lines were constructed, and the LNCaP cells were dispersed into a cell suspension (1 × 10^6^ cells/100 μL) with PBS. An amount of 0.2 mL of the cell suspension was then injected into the dorsal side of the nude mice (n = 10). The inoculated animals were housed in SPF-level animal cages. The tumor formation of nude mice was measured and recorded every 7 days, after which, a curve of the tumor growth was plotted. The tumor volume (V) was calculated using the formula: V = 1/2 (a × b^2^).

### Statistical analysis

All data were analyzed using the SPSS 21.0 software (IBM Corp., Armonk, NY, USA). Measurement data were expressed as mean ± standard deviation. Paired *t*-test was applied to compare paired data obeying normal distribution and homogeneity of variance between two groups, while unpaired *t*-test was used to compare unpaired data between two groups. Comparisons among multiple groups were analyzed by one-way analysis of variance (ANOVA), followed by Tukey’s post hoc test. Repeated measures ANOVA was applied to analyze the data at different time points during the experiment, followed by Bonferroni’s post hoc test. A value of *p *< 0.05 was considered to be statistically significant.

## Results

### LINC00908 is poorly expressed in PCa tissues and cell lines

**Fig. 1:**
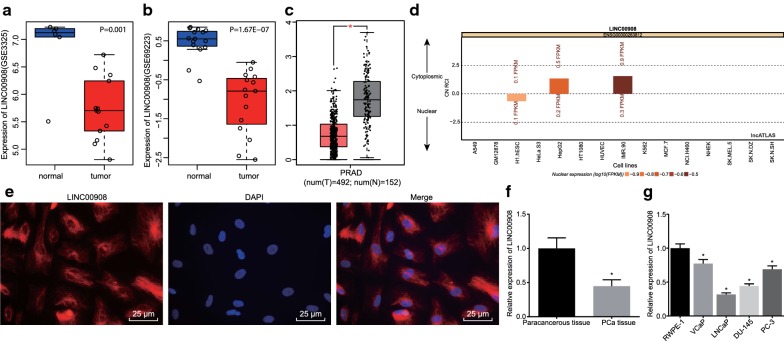
Low expression of LINC00908 in PCa tissues and cells. **a** The expression of LINC00908 in PCa-related microarray GSE3325 from the GEO database. **b** The expression of LINC00908 in PCa-related microarray GSE69223 from the GEO database. **c** The expression of LINC00908 detected in the GEPIA database. The abscissa represents the sample type and the ordinate represents the expression value; the red box plot represents the tumor sample, and the gray box plot represents the normal sample (* q < 0.01). **d** The localization of LINC00908 analyzed by the lncATLAS website (http://lncatlas.crg.eu/). **e** The subcellular localization of LINC00908 in cells detected by FISH assay (× 400). **f** LINC00908 expression in PCa tissues (n = 55) and paracancerous tissues (n = 55) determined by RT-qPCR, normalized to β-actin, *p < 0.05 vs. paracancerous tissues. **g** LINC00908 expression in RWPE-1, VCaP, LNCaP, DU-145, and PC-3 cell lines determined by RT-qPCR, normalized to β-actin, *p < 0.05 vs. RWPE-1 cell lines. The above results were measurement data, expressed as mean ± standard deviation, and *t*-test was used for data comparisons between two groups, while one-way ANOVA and Tukey’s post hoc test were used for comparisons among multiple groups. The cell experiment to measure LINC00908 expression in different cell lines was repeated three times

Firstly, differential analyses were performed on PCa-associated microarray datasets GSE3325 and GSE69223 (Fig. 1a, b) obtained from the GEO database and GEPIA database (http://gepia.cancer-pku.cn/index.html) (Fig. 1c), which revealed that LINC00908 was significantly down-regulated in PCa. Further analysis from the lncATLAS website (http://lncatlas.crg.eu/) indicated that LINC00908 was mainly localized in the cytoplasm (Fig. 1d), suggesting that LINC00908 could regulate the expression of miRNAs post-transcriptionally. As illustrated in Fig. 1e, the blue fluorescence represented the nucleus and red fluorescence represented LINC00908 expression. FISH demonstrated that LINC00908 was indeed mainly expressed in the cytoplasm. In addition, RT-qPCR results showed that the expression of LINC00908 in human PCa tissues was significantly lower compared to paracancerous tissues (*p *< 0.05, Fig. 1f). At the same time, the expression of LINC00908 in all four human PCa cell lines VCaP, LNCaP, DU-145, and PC-3 was significantly decreased compared to RWPE-1 cell lines, with a more pronounced decline in LNCaP cells (all *p* < 0.05, Fig. 1g). Therefore, the LNCaP cell lines were chosen for the subsequent experimentation. The above results indicated that LINC00908 was poorly expressed in PCa tissues and cells.

### Overexpression of LINC00908 inhibits PCa cell proliferation, migration, and invasion while inducing apoptosis

**Fig. 2:**
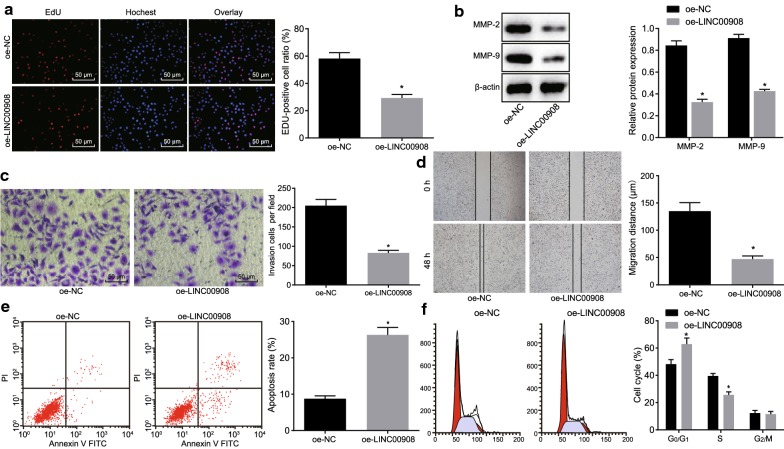
Overexpression of LINC00908 suppresses PCa cell proliferation, migration, and invasion while promoting apoptosis. LNCaP cells were treated with oe-LINC00908 or with oe-NC as the control. **a** The effect of LINC00908 on cell proliferation in each group (×200) detected by EdU assay. **b** The protein expression of MMP-2 and MMP-9 in cells measured by Western blot analysis, normalized to β-actin. **c** Cell invasion assessed by Transwell assay (×200). **d** Cell migration assessed by scratch test. **e** Cell apoptosis assessed by flow cytometry. **f** Cell cycle distribution assessed by flow cytometry. *p < 0.05 vs. cells treated with oe-NC. The results were measurement data, expressed as mean ± standard deviation, and the unpaired *t*-test was used for data between two groups. The cell experiment was repeated three times

Having identified the low expression of LINC00908 in PCa, we next focused on the role of LINC00908 in the biological functions of PCa cells. EdU assay displayed that the proliferation rate of LNCaP cells transfected with oe-LINC00908 was significantly lowered (*p* < 0.05, Fig. 2a). MMP-2 and MMP-9 are known as extracellular matrix metalloproteinases that can degrade type IV collagen and gelatin. Because the main component of extracellular matrix basement membrane is type IV collagen, MMP-2 and MMP-9 play a key role in the degradation of extracellular matrix and promotion of tumor invasion and metastasis [18, 19]. We therefore determined the expression of MMP-2 and MMP-9 using western blot analysis, which showed that the protein expression of MMP-2 and MMP-9 was remarkably down-regulated in LNCaP cells upon oe-LINC00908 treatment (*p* < 0.05, Fig. 2b), suggesting that LINC00908 could impede the invasion and migration of PCa cells. Scratch test and Transwell assay displayed that the migration and invasion abilities of oe-LINC00908-treated LNCaP cells was reduced (both *p* < 0.05, Fig. 2c, d). Flow cytometry showed that the apoptosis rate of LNCaP cells post oe-LINC00908 treatment was significantly increased with more cells arrested in the G0/G1 phase and fewer cells arrested in the S phase (*p* < 0.05, Fig. 2e, f). The above results indicated that the overexpression of LINC00908 could remarkably inhibit PCa cell proliferation, migration, and invasion, whereas promote apoptosis.

### LINC00908 competitively binds to miR-483-5p

**Fig. 3:**
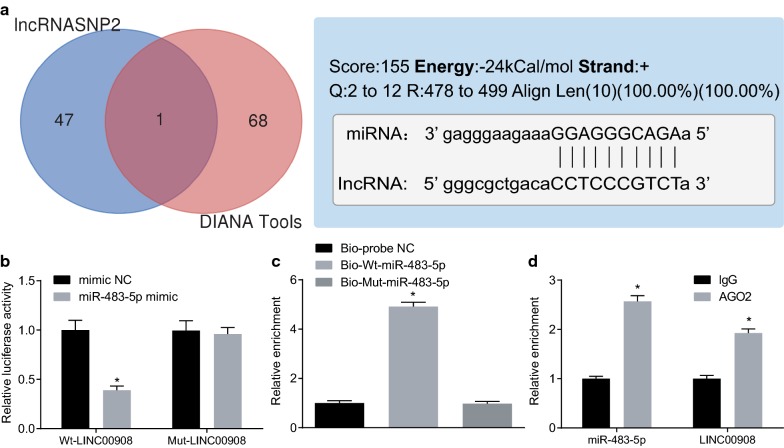
LINC00908 competitively binds to miR-483-5p in vitro. **a** The binding of LINC00908 to miR-483-5p analyzed by DIANA Tools (http://diana.imis.athena-innovation.gr/DianaTools/index.php?r=site/page&vie=software) and lncRNASNP2 database (http://bioinfo.life.hust.edu.cn/lncRNASNP#!/). The two circles in the figure respectively represent the predicted results from the lncRNASNP2 database and the DIANA database, and the middle part represents the intersecting miRNAs predicted from the two databases. **b** The relationship between LINC00908 and miR-483-5p verified by dual-luciferase reporter assay. *p < 0.05 vs. cells upon mimic NC treatment, **c** LINC00908 binding to miR-483-5p analyzed by RNA pull-down assay. *p < 0.05 vs. cells upon the Bio-probe NC treatment. **d** The binding relationship between LINC00908 and AGO2 detected by RIP. *p < 0.05 vs. cells upon the IgG treatment. The data were measurement data and expressed as the mean ± standard deviation. Unpaired *t*-test was used for data between two groups, while one-way ANOVA was used for data among multiple groups, followed by Tukey’s post hoc test. The cell experiment was repeated three times

Next, we continued to further understand the mechanism of action of LINC00908 in PCa. We first used DIANA Tools (http://diana.imis.athena-innovation.gr/DianaTools/index.php?r=site/page&vie=software) and lncRNASNP2 database (http://bioinfo.life.hust.edu.cn/lncRNASNP#!/) and found a binding relationship between miR-483-5p and LINC00908 (Fig. 3a). The dual-luciferase reporter assay further confirmed that the luciferase activity of the Wt-LINC00908 containing miR-483-5p binding site was inhibited by miR-483-5p mimic (*p* < 0.05), while the luciferase activity of Mut-LINC00908 was not inhibited (*p* > 0.05, Fig. 3b). In addition, RNA pull-down assay showed no significant difference in the enrichment of LINC00908 between the Bio-Mut-miR-483-5p and the Bio-probe NC treatment (*p* > 0.05), while LINC00908 enrichment was significantly increased by Bio-Wt-miR-483-5p (*p* < 0.05, Fig. 3c), indicating that Bio-Wt-miR-483-5p could promote the enrichment of LINC00908. RIP assay demonstrated that AGO2-bound LINC00908 was significantly increased compared to IgG (*p* < 0.05, Fig. 3d). The above results indicated that LINC00908 could competitively bind to miR-483-5p.

### LINC00908 enhances TSPYL5 expression via binding to miR-483-5p

**Fig. 4:**
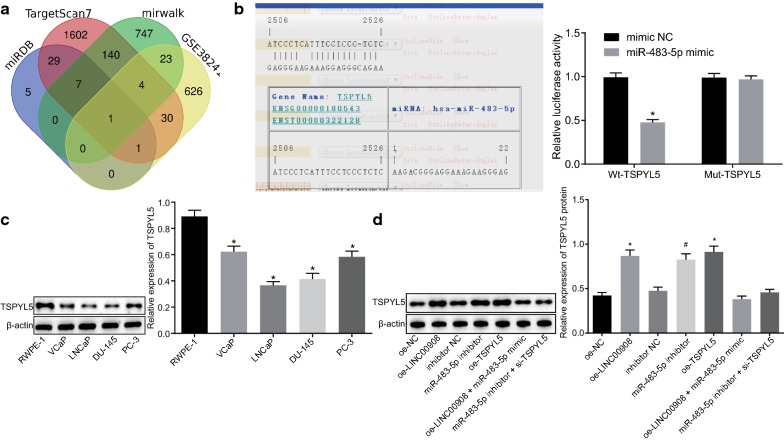
LINC00908 elevates the expression of TSPYL5 by binding to miR-483-5p. **a** TSPYL5 screened out using TargetScan (http://www.targetscan.org/vert_71/), miRDB (http://mirdb.org/miRDB/index.html), and mirwalk (http://mirwalk.umm.uni-heidelberg.de/) databases, in combination with the microarray GSE38241. The four ellipses plotted in the figure represent the predicted results from the TargetScan, database, miRDB and mirwalk databases as well as GSE38241 dataset respectively. The middle part represents the intersecting genes from them. **b** The luciferase activity of Wt-TSPYL5 or Mut-TSPYL5 in cells transfected with miR-483-5p mimic. *p < 0.05 vs. cells upon mimic NC treatment. **c** The expression of TSPYL5 in VCaP, LNCaP, DU-145, and PC-3 cells and normal RWPE-1 cell line detected by Western blot analysis, normalized to β-actin, * *p* < 0.05 vs. RWPE-1 cells. **d** The protein expression of TSPYL5 in LNCaP cells after the alteration of LINC00908, miR-483-5p and/or TSPYL5 determined by Western blot analysis, normalized to β-actin, *p < 0.05 vs. cells upon oe-NC treatment, ^#^*p* < 0.05 vs. cells upon inhibitor NC treatment. The data were measurement data and expressed as the mean ± standard deviation. Unpaired *t*-test was used for data between two groups, while one-way ANOVA was used for data among multiple groups, followed by Tukey’s post hoc test. The cell experiment was repeated three times

To identify the target gene for miR-483-5p, the target gene TSPYL5 was screened among the poorly expressed genes in the GSE38241 microarray according to the results from TargetScan (http://www.targetscan.org/vert_71/), miRDB (http://mirdb.org/miRDB/index.html), and mirwalk (http://mirwalk.umm.uni-heidelberg.de/) databases (Fig. 4a). Based on the screening results, we speculated that LINC00908 could regulate the expression of TSPYL5 by binding to miR-483-5p. Moreover, the RNA22 website (https://cm.jefferson.edu/rna22/) revealed a specific binding between miR-483-5p and TSPYL5. In addition, the luciferase activity of the Wt-TSPYL5 3′UTR was significantly decreased (*p* < 0.05), while that of Mut-TSPYL5 showed no significant difference in cells upon transfection with miR-483-5p mimic (*p* > 0.05, Fig. 4b), indicating that miR-483-5p could inhibit the expression of TSPYL5. Western blot analysis demonstrated that TSPYL5 was poorly expressed in PCa cells, with a more pronounced reduction in LNCaP cells, which was consistent with the results observed in LINC00908 expression (*p* < 0.05, Fig. 4c). In addition, the results from RT-qPCR and Western blot analysis showed that the expression of TSPYL5 was significantly up-regulated in cells treated by oe-LINC00908, miR-483-5p inhibitor or oe-TSPYL5 (all *p *< 0.05), while there was no significant difference between co-transfection with oe-LINC00908 and miR-483-5p mimic or the co-transfection with miR-483-5p inhibitor and si-TSPYL5 (*p* > 0.05, Fig. 4d). The above results indicated that LINC00908 could up-regulate the expression of TSPYL5 via binding to miR-483-5p.

### Silencing of miR-483-5p or overexpression of TSPYL5 inhibits proliferation, migration, and invasion of PCa cells while inducing apoptosis

**Fig. 5:**
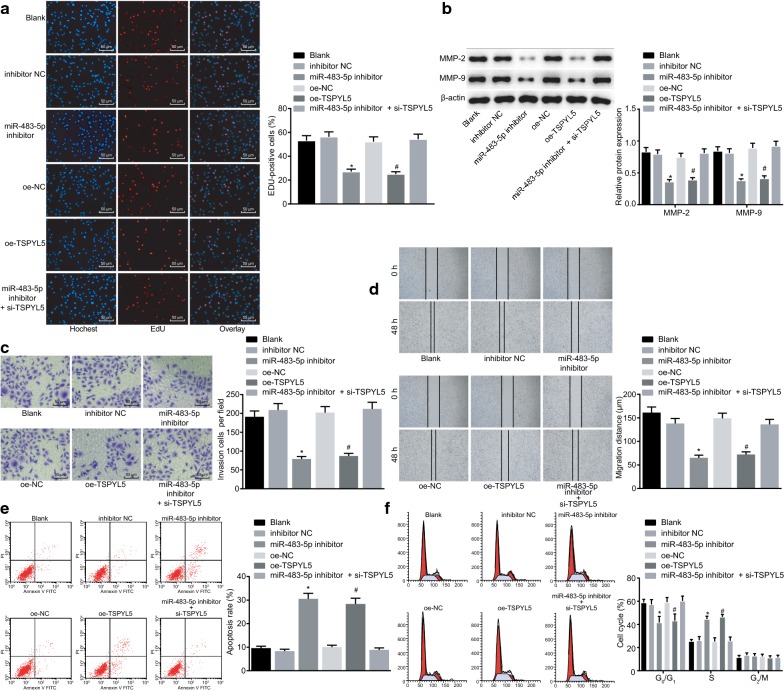
Silencing of miR-483-5p or overexpression of TSPYL5 exerts inhibitory effects on PCa cell proliferation, migration, and invasion, while stimulating apoptosis. LNCaP cells were treated with the miR-483-5p inhibitor, oe-TSPYL5, or miR-483-5p inhibitor and si-TSPYL5. **a** The effect of miR-483-5p and TSPYL5 on cell proliferation in each group detected by EdU assay (×200). **b** The protein expression of MMP-2 and MMP-9 measured by Western blot analysis, normalized to β-actin. **c** Cell invasion detected by Transwell assay (×200). **d** Cell migration detected by scratch test. **e** Cell apoptosis was detected by flow cytometry. **f** The cell cycle distribution measured by flow cytometry. *p < 0.05 vs. cells treated with inhibitor NC, and ^#^*p* < 0.05 vs. cells treated with oe-NC. The results were measurement data and expressed as mean ± standard deviation. One-way ANOVA was used for data among multiple groups, followed by Tukey’s post hoc test. The cell experiment was repeated three times

To explore the effect of miR-483-5p and TSPYL5 on the PCa development, a series of experiments were performed to determine the influence of miR-483-5p silencing or TSPYL5 overexpression on the cell proliferation, migration, invasion, and apoptosis. The results of the EdU assay revealed that there was no significant difference in cell proliferation rate between cells treated with miR-483-5p inhibitor and si-TSPYL5 and untreated cells (*p* > 0.05). The proliferation rate of LNCaP cells transfected with miR-483-5p inhibitor and oe-TSPYL5 was significantly decreased (both *p *< 0.05, Fig. 5a). Western blot analysis revealed no significant difference in the protein expression of MMP-2 and MMP-9 between cells co-transfected with miR-483-5p inhibitor and si-TSPYL5 and untreated cells (*p *> 0.05). The protein expression of MMP-2 and MMP-9 in miR-483-5p inhibitor- and oe-TSPYL5-treated cells was significantly down-regulated (all *p* < 0.05, Fig. 5b). Additionally, Scratch test and Transwell assay demonstrated no significant difference in migration and invasive abilities between cells co-transfected with miR-483-5p inhibitor and si-TSPYL5 and untreated cells (*p* > 0.05). Whereas, migration and invasive abilities were significantly reduced in cells transfected with miR-483-5p inhibitor and oe-TSPYL5 (both *p* < 0.05, Fig. 5c, d). Flow cytometry also illustrated no significant changes in apoptosis rate and cell cycle distribution between cells co-transfected with miR-483-5p inhibitor and si-TSPYL5 and untreated cells (*p* > 0.05). The apoptosis rate of miR-483-5p inhibitor- and oe-TSPYL5-treated cells was significantly increased, and a significant increase in G0/G1 phase-arrested cells but a significant decrease in S phase-arrested cells were observed (all *p* < 0.05, Fig. 5e, f). The collected data indicated that the inhibition of miR-483-5p could up-regulate TSPYL5 expression to inhibit PCa cell proliferation, invasion, and migration, while inducing cell apoptosis.

### LINC00908 inhibits the tumor growth of PCa in vivo

**Fig. 6:**
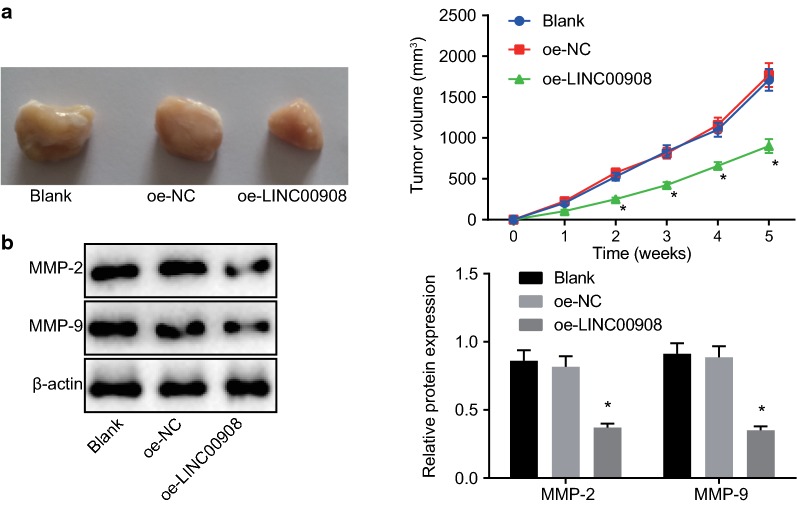
The restoration of LINC00908 hinders PCa tumor growth and reduces the expression of MMP-2 and MMP-9 in vivo. Nude mice with PCa were injected with oe-LINC00908 with oe-NC as the control. **a** Changes in tumor volume in nude mice bearing overexpression of LINC00908. **b** MMP-2 and MMP-9 expression in mice determined by Western blot analysis, normalized to β-actin. *p < 0.05 vs. mice injected with oe-NC-transfected cells or un-transfected cells. Data were measurement data, expressed as mean ± standard deviation, and one-way ANOVA was used for data comparisons among multiple groups followed by Tukey’s post hoc test. Repeated measures ANOVA was applied for data comparisons between groups at different time points with Bonferroni’s post hoc test. N = 10

Findings in nude mice showed that tumor growth was significantly inhibited in the mice injected with oe-LINC00908-transfected cells compared to nude mice injected with untransfected cells or oe-NC-transfected cells (*p* < 0.05, Fig. 6a). Western blot analysis was performed to measure the expression of downstream factors (MMP-2, and MMP-9) and the results showed that the protein expression of MMP-2 and MMP-9 was significantly diminished after the injection with oe-LINC00908-transfected cells (*p* < 0.05, Fig. 6b). The above results served to illustrate that overexpression of LINC00908 inhibited tumor growth in vivo.

## Discussion

Despite the advent of clinical biomarkers for PCa such as prostate-specific antigen, various drawbacks still cause inefficient diagnosis and prognosis of PCa. Therefore, further studies are necessary to improve the diagnosis, prognosis and therapeutic outcomes of PCa [20]. The current study aimed to investigate the role of LINC00908 in PCa involving TSPYL5, and uncovered that LINC00908 could potentially promote the expression of TSPYL5 by competitively binding to miR-483-5p, thus inhibiting invasion and migration of PCa cells.

Our findings suggested that LINC00908 was poorly expressed in PCa tissues and cells, while the overexpression of LINC00908 resulted in inhibited PCa cell proliferation, migration, and invasion, as well as accelerated apoptosis. The results can be supported by the evidence that various lncRNAs have been identified to be involved in the regulation of tumor initiation and growth in human cancers including PCa [21]. For instance, a previous study identified that lncRNA GASL1 was poorly expressed in PCa, while upregulation of GASL1 exerts an inhibitory effect on PCa cell growth [22]. Similarly, another lncRNA BRE-AS1 has been determined to be downregulated in PCa, and BRE-AS1 overexpression inhibits cell progression and enhances apoptosis of PCa cells [23]. In addition, evidence also demonstrates that lncRNA TINCR is poorly expressed in PCa cells and overexpression of TINCR suppresses cell invasion and migration as well as decreases proliferation in PCa [24]. LINC00844 has been documented to be decreased in malignant and metastatic PCa cells, and acts as a tumor suppressor in cancer progression and metastasis [25], which is in line with our findings. Furthermore, in vivo experimentation in our study verified that LINC00908 could inhibit the tumorigenic ability of mice. However, few researchers have studied the function of LINC00908 on cancer development.

Additionally, functional experiments in the current study manifested that LINC00908 reduced the proliferation and migration of PCa cells by downregulating miR-483-5p. This finding was consistent with the previous study where miRNA expression has been proved to be involved in the progression and development of multiple cancers including PCa [26, 27]. A prior study has demonstrated that miR-483-5p is upregulated in the cell-free urine fraction of patients with PCa [28]. A recent study has also revealed the high expression of miR-483-5p in PCa cell lines, and suppressing miR-483-5p contributes to the inhibition of PCa cell migration, invasion, and proliferation [15]. In addition, another study has also revealed that the upregulation of miR-438-5p results in tumor aggressiveness [29]. In parallel with the aforementioned studies, our findings imply that inhibition of miR-483-5p could attenuate the progression of PCa.

Furthermore, the current study discovered that miR-483-5p decreased expression of TSPYL5, thus promoting proliferation and migration of PCa cells. We further revealed that overexpression of TSPYL5 affected PCa progression in an inhibitory manner. Meanwhile, LINC00908 overexpression inhibited the proliferation, migration, and invasion of PCa cells via upregulation of TSPYL5 by binding to miR-483-5p, which was similar to the expression of MMP-2 and MMP-9. MMP-2 and MMP-9 are widely regarded as factors associated with cell migration and invasion in multiple types of tumors [30–32]. Moreover, another research highlighted TSPYL5 as a tumor inhibitor gene and was poorly expressed in PCa [17]. Similarly, previous research has demonstrated that decreasing TSPYL5 expression may influence ovarian cancer cell invasion and proliferation in a suppressive way [33].

## Conclusions

**Fig. 7:**
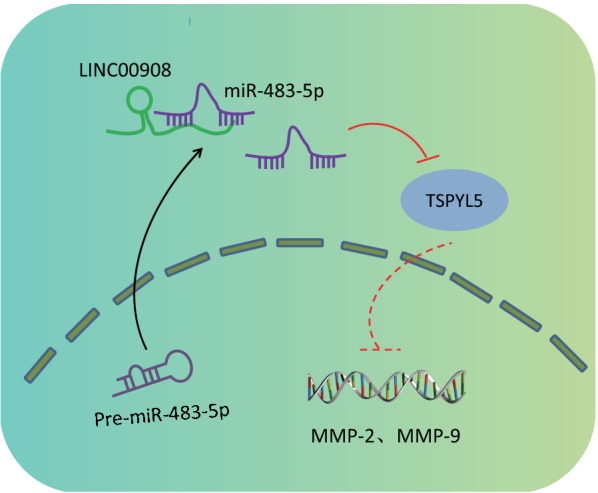
Schematic diagram depicts the regulatory mechanism of the LINC00908/miR-483-5p/TSPYL5 axis in PCa progression. LINC00908 competitively binds to miR-483-5p and up-regulates TSPYL5 expression, thereby inhibiting the expressions of MMP-2 and MMP-9, PCa cell proliferation, as well as promoting cell cycle arrest and apoptosis

In summary, our study demonstrated that LINC00908 was underexpressed in PCa, and LINC00908 increased TSPYL5 expression by competitively binding to miR-483-5p, thereby inhibiting invasion and migration of PCa cells (Fig. 7). These findings highlight LINC00908 as a novel biomarker for PCa prognosis, as well as a promising therapeutic target for patients with PCa. Nonetheless, our findings solely shed a light on the theoretical basis for the underlying mechanism in LINC00908 in PCa. Therefore, clinical experiments of the fully developed anti-cancer therapeutic agent should be perfected in the future studies. Moreover, further research is warranted in order to explore the specific mechanism on various molecules as well as signaling pathways underlying the progression and development of PCa.

## Data Availability

The datasets generated and/or analysed during the current study are available from the corresponding author on reasonable request.

## Abbreviations

PCa: prostate cancer
lncRNAs: long non-coding RNAs
miRNAs or miRs: microRNAs
TSPYL5: testis-specific protein Y-encoded-like 5
GEO: Gene Expression Omnibus
FISH: fluorescence in situ hybridization
RIP: RNA binding protein immunoprecipitation
EdU: 5-ethynyl-2′deoxyuridine
HCG11: human chorionic gonadotropin 11
GAS5: growth arrest-specific transcript 5
TNM: tumor-nodes-metastasis
FDR: false discovery rate
FC: fold change
RT-qPCR: reverse transcription quantitative polymerase chain reaction
NCBI: National Center for Biotechnology Information
RPMI: Roswell Park Memorial Institute
oe: overexpression
si: small interfering RNA
NC: negative control
PBS: phosphate-buffered saline
DAPI: 4′,6-diamidino-2-phenylindole
PBST: PBS-Tween-20
cDNA: complementary DNA
RIPA: radioimmunoprecipitation assay
SDS: sodium dodecyl sulfate
PAGE: polyacrylamide gel electrophoresis
MMP: matrix metalloproteinase
IgG: immunoglobulin G
ECL: enhanced chemiluminescence
HRP: horseradish peroxidase
UTR: untranslated region
AGO2: Argonaute 2
PI: propidium iodide
FITC: fluorescein isothiocyanate
FBS: fetal bovine serum
SPF: specific pathogen-free
ANOVA: analysis of variance
